# Development and implementation of a global Roche cell culture platform for production of monoclonal antibodies

**DOI:** 10.1186/1753-6561-7-S6-P24

**Published:** 2013-12-04

**Authors:** Thomas Tröbs, Sven Markert, Ulrike Caudill, Oliver Popp, Martin Gawlitzek, Masaru Shiratori, Chris Caffalette, Robert Shawley, Steve Meier, Abby Pynn, Wendy Hsu, Andy Lin

**Affiliations:** 1 Pharmaceutical Biotech Production & Development PTDE, Roche, 82377 Penzberg, Germany; 2 Pharma Research and Early Development pRED, Roche, 82377 Penzberg, Germany; 3 Early and Late Stage Cell Culture PTDU, Genentech, South San Francisco, CA 94061, USA

## Introduction

Roche and Genentech both developed their first platform cell culture process using chemically-defined media independently. This resulted in significantly different processes with regards to operations and media formulations. The decision was made to evaluate both and decide for one existing platform. Drivers and benefits of a single upstream cell culture platform were the maximization of flexibility with regard to process development, clinical and commercial manufacturing by execution of any process at any network facility with standard transfer effort and by minimization of component lists and raw material inventories across sites. Furthermore capturing benefits of improvements made by all sites funneled into a common knowledge base benefits the whole organization. And process characterization and validation data could be leveraged across the entire organization what means less resource expenditure for PC/PV.

The existing independent platforms were evaluated if there is a clear benefit in going forward with a given platform or certain aspects of a platform. The comparison consisted in a technical (cell culture performance, product quality, manufacturability) and a business case evaluation (product titer, timelines to launch, costs, IP. In result both platforms are capable of achieving sufficient titers for platform process (2-4 g/L) with acceptable product quality. There existed no major business driver to select one process over the other.

## Development

**Figure 1:**
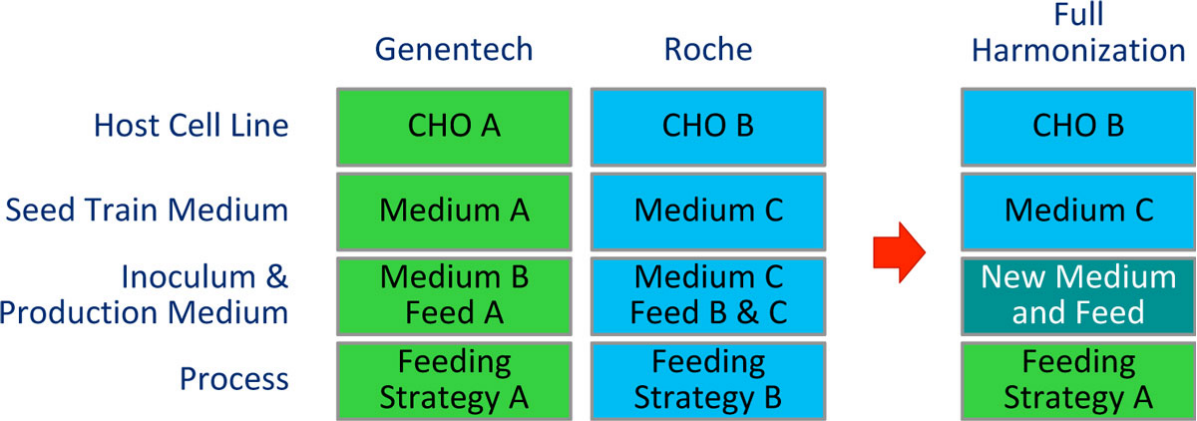
**Schematic diagram of major elements of the two legacy platforms and the optimization of medium and feed respective the leveraging of knowledge from two existing legacy platforms**.

For development of new basal and feed media knowledge from two legacy efforts was leveraged and so potential synergies and performance benefits could be achieved (Figure 1). Based on platform evaluation results, decision was made to harmonize existent CHO host cell line, seed train medium and feeding strategy (chosen from the two existing platforms).

## Results

The cell culture media and feed optimization strategy started with a paper exercise to compare existing in-house chemically defined media formulations and identify components/component groups for further evaluation. Subsequently identified conditions were screened in high-throughput cell culture systems (HTS-CC) to identify beneficial components and remove components that are not required. Optimized best cases were confirmed in 2L bioreactors with 6 model cell lines and the final process was up-scaled to pilot scale.

Promising results from HTS-CC media screening were confirmed in a 2L-bioreactor experiment. The new platform medium and feed were finalized after a series of 2L optimization experiments. The process was successful up-scaled to 250L single-use bioreactor (SUB) and 400L stainless steel bioreactor with two model cell lines. Growth and titer were comparable to 2L satellites.

In the course of the platform implementation four new GMP raw materials (dry powders and stock solution) were developed and tested. Raw material shelf life stability retesting and extension were initiated. Global specifications were established for equipment and site independent platform application and the applicability for global production units is given.

High temperature/short time treatment (HTST) compatibility was tested. The sterile hold for liquid media was initiated.

## Summary

New chemically defined platform media (basal and feed) were developed by leveraging data and knowledge from the two Genentech and Roche legacy platform processes, and through a series of experiments including high-throughput systems for cell culture, shake flasks, 2L bioreactors and pilot-scale bioreactors. An average increase in final titer of 30% was achieved compared to the two legacy platforms.

The final process resulted in product quality attributes (glycans, charge variants, size) that were comparable to historical data. No new variants were detected. The final and fully harmonized platform process is specified and implemented.

